# Correlates of functional ankle instability in children and adolescents with Charcot-Marie-Tooth disease

**DOI:** 10.1186/s13047-015-0118-1

**Published:** 2015-11-05

**Authors:** Kristy J. Rose, Claire E. Hiller, Melissa Mandarakas, Jacqueline Raymond, Kathryn Refshauge, Joshua Burns

**Affiliations:** Institute for Neuroscience and Muscle Research at The Children’s Hospital at Westmead, Sydney, NSW Australia; Arthritis and Musculoskeletal Research Group, Faculty of Health Sciences, The University of Sydney, Sydney, NSW Australia; Exercise Physiology and Nutrition Research Team, Faculty of Health Sciences, The University of Sydney, Sydney, NSW Australia; School of Physiotherapy, Faculty of Health Sciences, The University of Sydney, Sydney, NSW Australia

**Keywords:** Charcot-Marie-Tooth disease, Child, Adolescent, Functional ankle instability, Cumberland ankle instability tool, Foot posture

## Abstract

**Background:**

Functional ankle instability (FAI) is commonly reported by children and adolescents with Charcot-Marie-Tooth disease (CMT), however,, the specific variables associated with FAI remain unknown. An improved understanding of these variables may suggest interventions to improve ankle stability and possibly prevent the long-term complications associated with ankle instability in this population. The aim of this study was to therefore investigate the relationship between FAI and other functional, structural, anthropometric and demographic characteristics in a cross sectional sample of children and adolescents with CMT.

**Methods:**

Thirty children and adolescents with CMT aged 7–18 years were recruited from the Peripheral Neuropathy Clinics of a large tertiary paediatric hospital. Measures of FAI were obtained using the Cumberland Ankle Instability Tool (CAIT). Demographic and anthropometric data was also collected. Other variables collected included foot structure (Foot Posture Index), ankle range of motion (weight bearing lunge) and functional parameters (balance, timed motor function and falls). Descriptive statistics were calculated to characterise the participants. Pearson’s correlation coefficients were calculated to investigate the correlates of right and left FAI and demographic (age), anthropometric (height, weight, BMI), foot/ankle (foot structure and ankle flexibility) and functional parameters (balance task, timed motor function and falls frequency). Point biserial correlation was employed to correlate gender with right and left FAI.

**Results:**

All but one study participant (*n* = 29) reported moderate to severe bilateral FAI with females reporting significantly greater ankle instability than males. FAI was significantly associated with cavus foot structure (*r* = .69, *P* < .001), female gender (*r* = −.47, *P* < .001) and impaired balance (*r* = .50, *P* < .001).

**Conclusions:**

This study confirms FAI is common in children and adolescents with CMT. An examination of the correlates of FAI suggests interventions, which target balance, and normalise foot structure should be explored to evaluate whether they might help to improve ankle stability in this population.

## Background

The most common neuropathy of childhood, Charcot-Marie-Tooth disease (CMT), describes a group of clinically and genetically heterogeneous neuropathies characterised by a common phenotype of abnormal neurophysiology, absent tendon reflexes, distal sensory loss and progressive distal muscle weakness and atrophy [1]. Common clinical complaints from patients with CMT include muscle cramps, hand tremor [2] and problems with functional tasks [3]. Functional ankle instability (FAI) is frequently reported by children and young people with CMT. Indeed, one previous study investigating the foot and ankle manifestations of children with CMT type 1A found 72 % reported FAI [3]. FAI is a type of chronic ankle instability most frequently precipitated by an ankle sprain [4]. It refers to an individual’s perception that their ankle is weaker, less functional, more unstable and painful than before their initial ankle injury [3, 5]. To date, the largest body of research in FAI exists for the healthy adult population and indicates functional ankle instability can be debilitating, often leading to a wide spectrum of disability [6]. FAI has been reported to prevent otherwise healthy individuals from returning to their usual occupation and lead to changes to participation, including cessation of sporting and other occupational activities [6]. It is highly likely that the pre-existing complications of CMT such as foot deformity, sensory impairment and foot and ankle muscle weakness and imbalance may lead to even greater disability with FAI than in an otherwise healthy population.

There are a number of variables that could predispose individuals with CMT to FAI. In CMT, length-dependent degeneration of the tibial and peroneal nerves causes selective weakness and atrophy of the ankle evertors and dorsiflexors [7]. While the ankle invertors and plantarflexors are also affected, they remain relatively stronger and overpower the opposing muscle group [7], potentially predisposing the ankle to recurrent episodes of plantarflexion-inversion injury. High rates of cavovarus deformity have been reported in individuals undergoing foot and ankle surgery for chronic FAI [8]. Pes cavus is a frequently reported manifestation of CMT. The excessively supinated foot structure associated with the cavus foot type might predispose the ankle to recurrent episodes of plantarflexion and inversion injury, particularly when it is coupled with foot and ankle strength imbalance [8]. Previous research has shown poor proprioception and balance to be predictive of FAI in otherwise healthy individuals [9]. Demyelination and dysfunction of the sensory nerves is frequently reported in CMT [10]. Since sensory nerves are thought to play an important role in proprioception, the ability to establish a sense of joint position in space [11] and poor proprioception has been linked to FAI in healthy individuals [12] it is reasonable to suggest this could be the case in CMT.

An improved understanding of variables associated with FAI may suggest interventions to improve ankle stability and prevent the long-term complications associated with ankle instability in this population. Therefore, the aim of this study was to investigate the relationship between FAI and other functional, structural, anthropometric and demographic characteristics in children and adolescents with CMT.

## Methods

### Participants

Thirty children and adolescents aged 7–18 years with CMT were recruited through The Children’s Hospital at Westmead (Sydney, NSW, Australia). Children were included if they had a confirmed diagnosis of any type of CMT either by genetic testing or a confirmed genetic test in a first or second degree relative with a consistent clinical phenotype and confirmatory electrophysiological testing. Children were excluded if they had an acute lower limb injury or had undergone previous foot and ankle surgery. The study was approved by the Human Research and Ethics Committee of The Children’s Hospital at Westmead (Sydney, NSW, Australia). Informed consent was obtained from all study participants and/or their parents/guardians prior to the instigation of any study procedures.

### Foot and ankle measures

#### Functional ankle instability

Functional ankle instability (FAI) was assessed with the Cumberland Ankle Instability Tool (CAIT) [5]. The CAIT is a reliable and valid questionnaire with which to assess the severity of right and left FAI. The CAIT consists of nine statements regarding symptoms associated with FAI including ankle pain, whether the ankle feels unstable engaging in activities such as going down stairs, sporting activities, making sharp turns, jumping, hopping and standing on one leg. It also asks about recovery time following a typical incident of rolling over on the ankle and ability to stop the ankle from “rolling over”. The full version of the CAIT has been published previously [5].

The CAIT was administered to the study participants by an assessor, experienced in the administration of the questionnaire. The CAIT had only been validated for use with adults and previous research indicates that some adult respondents require clarification of some items. As we included participants as young as 7 years we felt it necessary to have someone with previous experience administer the questionnaire so they could provide clarification and assistance if required. The evaluator explained the questionnaire to the participants and their parents, read each of the nine statements, asking the participant to tick the box of the statement that best described their ankle status. Participants answered questions for the right ankle first, followed by the left ankle and were allowed to physically attempt any task if they were unsure of the answer. Some of the study participants wore in-shoe or ankle foot orthoses (AFOs). These participants were asked to answer the questions as if they were doing the activities without their orthoses. Scores were aggregated for each ankle to give a right and left CAIT score. The maximum possible CAIT score for each ankle is 30, indicating normal ankle stability, and the lowest score for each ankle is 0, indicating extreme ankle instability.

#### Ankle range of motion

To determine if lower limb joint laxity is associated with FAI, ankle range of motion was measured. Measures of ankle dorsiflexion range of motion were obtained bilaterally using the weight-bearing lunge test [13]. To conduct the lunge test, participants stood with one foot perpendicular to a wall and were asked to lunge forward towards the wall, using the wall for support if required. The assessor assisted to move the foot progressively further away from the wall until the maximum range of ankle dorsiflexion had been obtained without the heel lifting off the ground. The angle of the tibial shaft from vertical was then measured using a digital inclinometer (Baseline®, Fabrication Enterprises Inc, New York, USA). Pronation and supination of the subtalar and midtarsal joints was limited by ensuring the foot was positioned perpendicular to the wall and that the participants lunged directly over the midline of the foot (second toe). The lunge test has excellent reliability in children and young adults [14].

#### Foot structure

Foot structure was assessed using the Foot Posture Index (FPI) to test the theory that children may be more vulnerable to FAI because of an already inverted position of the ankle that a pes cavus foot posture may impose [15]. The FPI is a weight-bearing, criterion-based observational rating system, which allocates a score between −2 and +2 to each of six criteria related to foot structure (talar head palpation, supra- and infra- malleolar curvature, calcaneal frontal plane position, prominence in the region of the talonavicular joint, congruence of the medial longitudinal arch, and abduction/adduction of the forefoot on the rearfoot). Scores for each of the six criteria are aggregated and range from −12 (extremely supinated/pes cavus foot structure) to +12 (extremely pronated/pes planus foot structure). Foot type was categorised as pes cavus (FPI score = −1 to −12); normal (FPI score = 0 to +5); and planus (FPI score = +6 to 12). The FPI has proven reliability and construct validity as well as published normative values, for children and young adults [16, 17].

### Functional measures

#### Balance

Balance training is a common treatment method for individuals with FAI [18]. To determine if sensory effects of CMT impact ankle instability, balance was assessed using three tasks of increasing difficulty taken from the Berg Balance Scale [19]. These tasks included the amount of time (up to 30 s) the participant could maintain standing with the medial borders of the feet touching, standing with the big toe of one foot beside the heel of the other foot and standing with the toes of one foot placed directly behind the heel of the other foot (tandem stance).

#### Timed motor function

To assess functional tasks that may be impacted by FAI, three timed tests of motor function were assessed including time taken to walk up and down a standard flight of 10 stairs, time to stand from a chair (adjusted to a height where the participant’s hips and knees were at 90° of flexion) and time taken to walk 10 m at the participant’s fastest and self-selected speed. All tasks have previously established reliability and sensitivity in children and young people with neuromuscular disorders [20].

#### Falls

Falls are commonly reported in CMT and may be a result of instability at the ankle joint [3]. To observe any relationship between falls and FAI, participants were asked to keep a diary the week preceding the study visit where they recorded the number of falls to the ground they experienced each day. The number of falls recorded in the diary were added and used for analysis.

### Statistical Methods

Descriptive statistics were calculated to characterise the participants and normality of the data distribution was assessed. Pearson’s correlation coefficients were used to examine the relationships between right and left FAI and demographic (age), anthropometric (height, weight, body mass index), foot/ankle (foot posture and ankle dorsiflexion range of motion) and functional parameters (three balance tasks, stair ascent, walking at fastest and self-selected speeds and falls frequency). Point biserial correlation was employed to examine the relationship between male/female gender and right and left FAI.

## Results

### Participants

The physical characteristics of the 30 study participants with CMT aged 7–18 years are presented in Table 1. There were 14 male and 16 female participants. The majority of study participants had CMT Type 1A (74 %), followed by X-linked CMT (17 %), Spinal CMT (3 %), Dejerine-Sottas Syndrome (3 %) and CMT Type 2 (3 %). One child had attention-deficit hyperactivity disorder (ADHD Whilst attention was difficult to maintain, the assessor was experienced and able to obtain high-quality data, thus participation was not limited in any way. No further co-morbidities were reported for the other study participants. All of the children were able to walk independently. The child with Dejerine-Sottas-Syndrome used a power wheelchair for long distance mobility but walked short distances independently, hence was able to participate in all test items and report on ankle function. None of the other study participants used a mobility aid. Five of the children wore in-shoe orthoses on a daily basis. Three children wore articulated ankle-foot-orthoses (AFOs) on occasions when they felt their balance would be challenged, such as during sport. Two children wore hinged AFOs on a daily basis.

**Table 1:** Physical characteristics of the 30 study participants

Physical Characteristics	All	Males	Females
	(*N* = 30)	(*N* = 14)	(*N* = 16)
Age (years)	11 (3), 7–18	11 (3), 7–18	11 (3), 7–16
Height (cm)	141 (17), 115–176	143 (19), 122–176	139 (16), 115–162
Weight (kg)	38 (16), 22–91	41 (19), 13–30	36 (12), 22–57
Body Mass Index (kg/m^2^)	19 (4), 13–30	19 (4), 13–30	18 (3), 15–23
CMT sub-type, n (%)			
CMT1A	22 (74)	8 (57)	14 (88)
CMTX	5 (17)	4 (29)	1 (6)
Dejerine-Sottas Syndrome	1 (3)	0	1 (6)
Spinal CMT	1 (3)	1 (7)	0
CMT2	1 (3)	1 (7)	0
Foot Posture Index Score right	−1 (5), −11 to +8	0.1 (5), −8 to +8	−2 (5), −11 to +6
Foot Posture Index Score left	−0.3 (5), −10 to +11	0.8 (6), −8 to +11	−1 (5), −10 to +5
Ankle range of motion right (^0^)	16 (6), 2–29	17 (6), 8–28	16 (6), 2–29
Ankle range of motion left (^0^)	17 (6), 2–29	17 (6), 5–25	16 (7),–3–23
Ankle instability right (CAIT Score)	18 (7), 5–30	21 (7), 5–30	15 (5), 5–25*
Ankle instability left (CAIT Score)	18 (7), 5–28	21 (7), 5–28	16 (5), 5–25*
Balance–feet side by side (seconds)	28 (7), 2–30	30 (0), 30–30	26 (9), 2–30
Balance–big toe next to heel (seconds)	25 (9),1–30	28 (6), 7–30	23 (11), 1–30
Balance–heel in front of toes (seconds)	22 (11), 1–30	21 (10), 2–30	22 (11), 1–30
Stair ascent (seconds)	14 (3), 8–21	14 (3), 9–21	13 (3), 8–17
Sit to stand (seconds)	1 (0.4), 0.4–2	1 (0.3), 0.4–2	1 (0.4), 1–2
Self–selected walking speed (seconds)	9 (3), 6–25	9 (2), 7–13	9 (4), 6–25
Fastest walking speed (seconds)	6 (3), 4–24	5 (0.6), 4–6	7 (5), 4–24
Falls (number/past week)	7 (10), 0–50	9 (13), 0–50	5 (5), 0–16

Values are mean (standard deviation), range except for gender, which is expressed as a ratio and CMT sub-type, which is expressed as a percentage

*Abbreviations: CMT1A* Charcot-Marie-Tooth disease Type 1A, *CMTX* X-Linked Charcot-Marie-Tooth disease, *CMT2* Charcot-Marie-Tooth disease Type 2, *CAIT* Cumberland Ankle Instability Tool

*Significant gender difference (*P* < .05)

### Foot, ankle and functional variables

Table 1 presents foot, ankle and functional variables for the study participants with CMT. Weight-bearing ankle dorsiflexion range of motion was limited bilaterally in all participants (≤25°), much lower than referenced norms for weight bearing ankle dorsiflexion range of motion [13]. Foot posture ranged from pes cavus to pes planus: FPI score of −11 to +8 (mean −1, SD 5) on the right and −10 to +11 (mean −0.3, SD 5) on the left. Thirteen participants had FPI scores indicative of cavus foot posture. All but one study participant had CAIT scores indicative of bilateral FAI (mean 16, SD 6 on the right and mean 17, SD 6 on the left). These scores are well below the cut-off score of 27 that indicates the presence of FAI [5].

Female participants reported significantly lower CAIT scores than male participants for both right (*t* = 2.807, *P* < .05) and left (*t* = 2.279, *P* < .05) ankles, indicating greater instability in girls. No significant gender differences (*P* < .05) were observed for age, anthropometric variables (height, weight, body mass index), dorsiflexion range of motion, foot posture, balance activities (standing with feet together, standing with the toes of one foot next to the heel of the opposite foot and standing with the toes of one foot behind the heel of the opposite foot) or functional parameters (walking at self-selected or fastest speeds, sit-to-stand and stair ascent). There were no significant right-to-left sided differences (*P* < .05) for measures of foot posture, dorsiflexion range of motion or FAI.

### Variables associated with ankle instability

Pearson’s *r* correlation coefficients revealed a number of explanatory variables that were significantly associated with right and left FAI (Table 2). Factors significantly associated with right FAI included female gender (*r* = −.47, *P* < .001), right cavus foot posture (*r* = .69, *P* < .001), toe-to-heel balance (*r* = .50, *P* < .001) and contralateral ankle instability (*r* = .97, *P* < .001). Factors associated with left FAI included female gender (*r* = .40, *P* < .05), left cavus foot posture (*r* = .73, *P* < .001), toe-to-heel balance (*r* = .47, *P* < .001) and contralateral FAI (*r* = .97, *P* < .001).

**Table 2:** Associations between right and left functional ankle instability, and demographics foot and ankle characteristics, motor function and balance in 30 children aged 7–18 with Charcot-Marie-Tooth disease

Ankle instability	Age (years)	Gender	Balance (1)	Balance (2)	Balance (3)	Falls	Walk (fastest)	STS (secs)	Stairs (secs)	ROM (R)	ROM (L)	FPI (R)	FPI (L)
CAIT (R)	-.09	-.47^**^	.32	.50^**^	-.20	.17	-.16	-.06	-.02	.14	.18	.69^**^	.744^**^
CAIT (L)	-.16	-.40^*^	.32	.47^**^	-.23	.15	-.16	-.10	.01	.17	.21	.68^*^	.725^**^

Values are Pearson’s correlation coefficients or point biserial correlation coefficients (gender): ^*^ *P* < .05 and ^**^ *P* < .01

*Abbreviations*: *CAIT* Cumberland Ankle Instability Tool, *BMI* Body Mass Index, *PSFS* Patient Specific Function Scale, *Balance 1* Standing with medial borders of feet touching, *Balance 2* Standing with the toes of one foot next to the heel of the opposite foot, *Balance 3* Standing with the toes of one foot behind the heel of the opposite foot, *ST*S Sit to stand, *ROM* Ankle dorsiflexion range of motion, *FPI* Foot posture index

## Discussion

The results of this study confirm that many children and adolescents with CMT experience FAI. All but one participant in this study had CAIT scores below 27, the cut off point for determining whether an individual has FAI [5]. Recent research in adults suggests a lower cut off score of 25 as an indication of FAI [21]. When applying this cut off score to our study, 80 % of participants would be considered to have FAI. CAIT scores ranged from 5–30 bilaterally indicating some of the participants had severe FAI. Interestingly, females reported significantly greater FAI than males. This gender difference was not reported in a study of 236 otherwise healthy individuals with FAI aged 12 to 65 [5]. This finding is worthy of future investigation.

This study was limited in the number of variables examined as we did not assess joint hypermobility. It is well acknowledged joint hypermobility is more prevalent in females than males [22, 23], and that there is a higher incidence of musculoskeletal problems such as ankle sprain in individuals with general joint hypermobility [23]. In CMT, the addition of joint hypermobility to pre-existing foot and ankle muscle imbalance, cavus foot posture and/or impaired sensorimotor control may have led to females experiencing more incidents of rolling or twisting the ankle, causing more severe FAI, as multiple questions in the CAIT query how participants feel when they roll or twist the ankle.

Although the CAIT has been administered in otherwise healthy young people and adults with FAI, aged 12–65 years, this was the first time the CAIT has been administered to a paediatric population with foot and ankle pathology. While it has been shown that young children can reliably answer questions regarding the impact of disease on their life [24], we had a trained assessor administer the questionnaire and provide clarification and assistance for the younger participants if required. Although the assessor felt most children understood the questions and had good insight into the extent of their FAI they found some of the younger children required clarification and assistance with some items. As a response to this limitation, authors have since validated a paediatric version of the CAIT – the Cumberland Ankle Instability Tool – *Youth* (the CAITY), to allow children to answer the questionnaire alone, without influence from a third party (parent or clinician) [25].

Interestingly, while pes cavus is often reported as a hallmark feature of CMT we found the study participants to have a variety of foot postures. Only 13/30 of the participants had a cavus foot structure (FPI score = −1 to −12). It is important to note that the majority of research to date has been in adult patients with CMT and foot structure could evolve to a more cavus posture later in life, especially during periods of musculoskeletal growth. Despite only 13 participants having cavus foot posture we found a strong relationship between FAI and the cavus foot with participants exhibiting more severe cavus foot posture experiencing greater ankle instability. The strong relationship between foot posture and ankle instability suggests interventions that aim to realign foot posture, may also improve FAI. Interventions such as foot orthoses [26] and orthopaedic surgery [27] have been suggested to influence the alignment and biomechanics of pes cavus in CMT. It would be worth focusing future research efforts to determine whether interventions that target foot posture also improve FAI in CMT. Indeed, a recent publication investigating health-related quality of life in children and young adults with CMT reported cavus foot posture is significantly related to reduced health-related quality of life [18], indicating it is a problem worthy of attention.

We also found a strong relationship between one of the more challenging balance tasks (time to stand with the toes of one foot behind the heel of the opposite foot) and FAI. Previous research has shown poor balance to be a predictor of recurrent ankle sprain [28]. A prior publication in a larger number of children with CMT1A found lower balance scores on Bruininks-Oseretsky Test of Motor Proficiency, 2^nd^ Ed. compared with age-equivalent norms [3]. It may be that balance retraining might also lead to improvements in ankle stability in CMT. Indeed, research suggests interventions which aim to retrain balance are effective in improving function and postural control in otherwise healthy individuals with FAI [28].

## Conclusions

This study indicates FAI is a common problem in children and adolescents with CMT. Future research should now focus on the development and validation of a paediatric version of the CAIT and whether interventions that normalise foot structure or improve balance also result in improved ankle stability for children and young adults with CMT.
